# Extirpation of a Pedunculated Fibro-myoma of the Uterus; Recidive Showing All the Characteristics of Sarcoma

**Published:** 1879-10

**Authors:** O. Stroinski

**Affiliations:** Chicago


### ARTICLE V.

### EXTIRPATION OF A PEDUNCULATED FIBRO-MYOMA OF THE UTERUS; RECIDIVE SHOWING ALL THE CHARACTERISTICS OF SARCOMA. By O. STROINSKI, M.D., Chicago.

Mrs. A. K., aged 28 years, German, married, was well developed and free from any hereditary taint. She had menstruated since her 17th year, and regularly though scantily until May, 1875, when, without any known cause, the flow became very profuse, and was followed by a copious watery discharge from the vagina. In December, 1875, she was married to a man whose first wife died of phthisis. The man never had syphilis, but was a confirmed toper. After her marriage, the metrorrhagia became more profuse, the leucorrhœa persistent; and a violent pain upon the top of her head, very often deprived her of rest and sleep. Totally unable to attend her household and exceedingly anæmic, the woman consulted me in July, 1877. Upon examination of the patient, who had a pronounced *facies uterina*, I found a marked ante-flexion of the uterus, its fundus being 1½ inches below and to the right side of the umbilicus. The abdomen was much enlarged. The cervical portion of the uterus was hypertrophied; but by gentle pressure, the index finger could be passed through the internal os, beyond which it met a globular mobile tumor. The sound could be passed completely around the tumor, which was perfectly movable and attached to the anterior wall of the uterus near the fundus. The next day, after fully dilating the os uteri, I drew the tumor down through the os by means of the forceps and cut it off with the écraseur. The hæmorrhage was very trifling, and the uterus, washed out with carbolized water, contracted very readily. The patient made a rapid recovery. The excised pear-shaped tumor measured 9 cm. in length; its lower broader portion had a diameter of 7 cm.; its peduncle, 2½ cm.; it had no capsule, but possessed a very dense, firm texture, and was of a yellowish white color, with a few reddish streaks (muscular fibres). The microscope disclosed remnants of mucous membrane at the periphery of the tumor, and its substance composed of connective tissue with well developed fibers and large nuclei; blood vessels or clusters of cells could not be found. The result of the microscopic examinations enabled me to pronounce the tumor a benign fibroma, or with regard to the few muscular fibers, fibro-myoma.

Mrs. K. continued to enjoy good health, and to menstruate regularly until the end of last year. In February last, she called at my office to show me a lump of a fœtid, gray, friable substance which on the previous day had passed from her with a great deal of blood. She told me that since December of last year she had had profuse menstruations, followed by a mucous discharge which was attended by a violent burning in the abdomen. I found the os uteri patulous; the forefinger could pass easily and met behind the os internum a nodular growth of a soft consistence, evidently filling the whole cavity of the uterus. In order to make a more careful palpation of the tumor, I pressed the uterus downward; but a very profuse hæmorrhage ensued, so that the patient fainted. An injection of a solution of perchloride of iron stopped the bleeding. I made an appointment with the patient for the removal of the tumor — which I considered malignant — on the following day; but on the evening of the same day I was sent for. The woman had violent pains, attended by profuse hæmorrhage and putrid discharge. In the vagina I found a tumor of the size of a goose egg, which I removed at once. The tumor behind the os internum had disappeared; but smaller and larger pieces were being expelled continuously. With the finger nail and the curette I carefully removed the remnants of the growth from the uterine wall. The bleeding was arrested by injecting a solution of iron; but the uterus contracted but little. In its posterior wall I detected a small tumor projecting into the uterine cavity. In the course of six weeks it grew from the size of a hazelnut to that of a walnut. During the same time cauliflower excrescences sprouted up from the seat of the first tumor and rapidly grew through the os into vagina. The removal by the écraseur and curette was attended by considerable hæmorrhage. They grew again and in a very short time filled the whole vagina, until death relieved the cachectic patient on July 21, 1879.

The post mortem examination disclosed sarcomatous tumors in the left lung and pleura, in the vagina and in the cellular tissues of the pelvis. In the abdominal cavity was much serum, the lower portion of the omentum thickened and adherent to the abdominal wall. The lower part of the abdominal cavity was occupied by a tumor which looked very much like a uterus in the fourth month of pregnancy with the frontal wall removed and the placenta exposed to the eye. This tumor was 11 cm. in length, and proved to be the enlarged uterus filled with a soft sarcoma which had deeply penetrated into the muscular wall of the womb. The anterior superior wall — where the fibroma had been removed — was perforated and the left tube and ovary were involved by the sarcomatous growth. It also affected the peritoneal coat of the bladder, which was thickened and intimately blended with the morbid growth. The tumor was composed of several irregular lobes, some of which were soft and very vascular, while others were of a firmer texture.

The microscope revealed a great variety of elements; healthy muscular fibers, tracts of epithelial cells growing into them; round cells, irregular cells and giant cells imbedded in a homogeneous plasma.

The tumor which was removed by spontaneous expulsion, had the size of a goose egg, and was pretty vascular; its cut surface showed a grey red periphery, and a white center. The latter was composed altogether of connective tissue, with many nuclei. The peripheral zone exhibited all stages of vivid cell proliferation and numerous clusters of round and fusiform cells.

I have given this minute description of the case, because several cases of spontaneous expulsion of tumor from the uterus which were reported in medical journals, were incorrectly interpreted; and also for the reason that this case clearly shows the development of the rare uterine sarcoma. We recognize three distinct phases in its development: (1.) Growth of a fibroid tumor (here from unknown cause). (2.) Growth of a fibro-sarcoma (here probably due to infection through carcinomatous juice). (3.) Formation of a diffuse sarcoma by infection of the uterine mucous membrane through the secretions of a sarcomatous ulcer. As far as I know, this is the first case in which the microscope has shown the recidive of a benign growth to be a malignant tumor; and, I believe, those cases reported as successful extirpations of sarcoma were fibroid tumors in a state of inflammation and decomposition. Recurrent fibroids have often been observed; more recently a number of cases of sarcoma have been reported, the most interesting are those of Hegar,* Frankenhaeuser† (by Rogivne), Gusserow,‡ Simson,§ Chambers,‖ Chrobak,¶ Rabl-Reckhard,** Clay,†† G. Thomas,‡‡ Winkel,§§ etc.

---

HYPODERMIC INJECTION OF MORPHIA. — Dr. H. H. Kane, of New York city, who has for some time past been collecting statistics on the hypodermic injection of morphia, would consider it a great favor if members of the profession, who see this and have had experience with the instrument, will answer the following questions:

1. What is your usual dose?
2. Do you use it alone or with atropia?
3. What is the largest amount you have ever administered?
4. Have you had inflammation or abscess at the point of puncture?
5. Have you had any deaths or accidents caused by this instrument?
6. Do you know of any cases of opium habit thus contracted?

Where there has been an autopsy (5) please state the fact and the results obtained therefrom. All communications will be considered strictly confidential, the writer's name being used only when he gives his full consent thereto. Address all letters to Dr. H. H. Kane, 366 Bleecker street, New York.

---

* Archiv fuer Gynæk, Band II.

† Du sarcome de l'utérus, Zuerich, 1876.

‡ Archiv f. Gynæk, und Handbuch der Frauenkr, 1878.

§ Sarcoma uteri, Edinburg Med. Jour., 1876.

‖ Cas d'epithéliome du corps de l'uterus.

¶ Archiv f. Gynæk, IV.

** Rabl Rueckhard, Beitr. zur Geburtsh u. Gynæk, I.

†† On diffuse sarcoma of the uterus, Lancet, 1877.

‡‡ Sarcoma of the uterus, New York Journal of Obstetrics, March 17.

§§ Archiv f. Gynæk. III.

